# The incidence of upper respiratory infections in children is related to the concentration of vanadium in indoor dust aggregates

**DOI:** 10.3389/fpubh.2024.1339755

**Published:** 2024-03-21

**Authors:** Nina Prokopciuk, Vaida Taminskiene, Laimute Vaideliene, Izabele Juskiene, Vitalija Svist, Indre Valiulyte, Vaidotas Valskys, Roberta Valskiene, Algirdas Valiulis, Tomas Aukstikalnis, Lukas Vaidelys, Mindaugas Butikis, Jolita Norkuniene, Nikolaj Tarasiuk, Arunas Valiulis

**Affiliations:** ^1^Clinic of Children’s Diseases, Faculty of Medicine, Institute of Clinical Medicine, Vilnius University, Vilnius, Lithuania; ^2^Department of Pathology and Forensic Medicine, Faculty of Medicine, Institute of Biomedical Sciences, Vilnius University, Vilnius, Lithuania; ^3^Human Ecology Multidisciplinary Research Group, Department of Public Health, Faculty of Medicine, Institute of Health Sciences, Vilnius University, Vilnius, Lithuania; ^4^Clinic of Children’s Diseases, Medical Academy, Lithuanian University of Health Sciences, Kaunas, Lithuania; ^5^Faculty of Medicine, Vilnius University, Vilnius, Lithuania; ^6^Kantonsspital Münsterlingen, Münsterlingen, Switzerland; ^7^Institute of Biosciences, Life Sciences Center, Vilnius University, Vilnius, Lithuania; ^8^Laboratory of Ecotoxicology, Nature Research Centre, Vilnius, Lithuania; ^9^Department of Rehabilitation, Physical and Sports Medicine, Institute of Health Sciences, Faculty of Medicine, Vilnius University, Vilnius, Lithuania; ^10^Department of Mathematical Statistics, Vilnius Gediminas Technical University, Vilnius, Lithuania; ^11^Clinic of Asthma, Allergy and Chronic Respiratory Diseases, Vilnius, Lithuania

**Keywords:** dust aggregates, microelemental composition, vanadium, respiratory infections, primary school, children

## Abstract

**Background:**

It has been reported that the disease-initiated and disease-mediated effects of aerosol pollutants can be related to concentration, site of deposition, duration of exposure, as well as the specific chemical composition of pollutants.

**Objectives:**

To investigate the microelemental composition of dust aggregates in primary schools of Vilnius and determine trace elements related to acute upper respiratory infections among 6-to 11-year-old children.

**Methods:**

Microelemental analysis of aerosol pollution was performed using dust samples collected in the classrooms of 11 primary schools in Vilnius from 2016 to 2020. Sites included areas of its natural accumulation behind the radiator heaters and from the surface of high cupboards. The concentrations of heavy metals (Pb, W, Sb, Sn, Zr, Zn, Cu, Ni, Mn, Cr, V, and As) in dust samples were analyzed using a SPECTRO XEPOS spectrometer. The annual incidence rates of respiratory diseases in children of each school were calculated based on data from medical records.

**Results:**

The mean annual incidence of physician-diagnosed acute upper respiratory infections (J00-J06 according to ICD-10A) among younger school-age children was between 25.1 and 71.3% per school. A significant correlation was found between vanadium concentration and the number of episodes of acute upper respiratory infections during each study year from 2016 to 2020. The lowest was r = 0.67 (*p* = 0.024), and the highest was r = 0.82 (*p* = 0.002). The concentration of vanadium in the samples of dust aggregates varied from 12.7 to 52.1 parts per million (ppm). No significant correlations between the other trace elements and the incidence of upper respiratory infections were found, which could be caused by a small number of study schools and relatively low concentrations of other heavy metals found in the samples of indoor dust aggregates.

**Conclusion:**

A significant and replicable correlation was found between the concentration of vanadium in the samples of natural dust aggregates collected in primary schools and the incidence of acute upper respiratory infections in children. Monitoring the concentration of heavy metals in the indoor environment can be an important instrument for the prevention and control of respiratory morbidity in children.

## 1 Introduction

Children are facing an increased number of health issues due to the deterioration of air quality (1, 2). They are particularly vulnerable to air pollution, especially during periods of faster growth and development (3–5). Greater permeability of respiratory epithelium and immature defense mechanisms can also play an important role. The primary school is a relatively new environment for children in terms of environmental pollution (6). Children spend more time at school (up to 4–8 h usually in the same classroom) than anywhere else except their own home. School is a new environment for these children in terms of environmental pollution. Prior research has focused mainly on the mass concentration of particulate matter PM_2.5_ (particles equal to or less than 2.5 μm) and PM_10_ (particles equal to or less than 10 μm) (7–12). However, we recently found an important role of the accumulation mode (0.3–1.0 μm) of aerosol particles in asthma morbidity among younger (6–11 years) school-age children (13). It was earlier reported that the effects of inhaled aerosols depend on the size of the particles, concentration, duration of exposure, place of precipitation in the respiratory tract as well as their specific chemical composition (14). Heavy metals can impair important biochemical processes posing a threat to human health, plant growth, and animal life (15, 16). They can also cause toxicity in certain organs of the human body, such as nephrotoxicity, neurotoxicity, hepatotoxicity, skin toxicity and cardiovascular toxicity. This can have adverse effects of the physical growth of children and a deleterious impact on bronchial epithelial cells, among other harmful results (17). Aerosol particles can coagulate and deposit in the form of dust; thus, it is most convenient to study the microelemental composition of the aerosols by collecting dust samples where they naturally accumulate. Such places in the classrooms not usually subjected to wet cleaning are the surfaces of high cupboards and places behind the radiator heaters where aerosol particles are deposited due to thermophoretic forces (18).

The aims of our study were to analyze the microelemental composition of dust and their concentration in the samples of natural dust aggregates taken from Vilnius primary schools and to determine which trace elements can be related to the incidence of acute upper respiratory infections in younger school-age children.

## 2 Materials and methods

### 2.1 Description of studied schools

**Figure 1:**
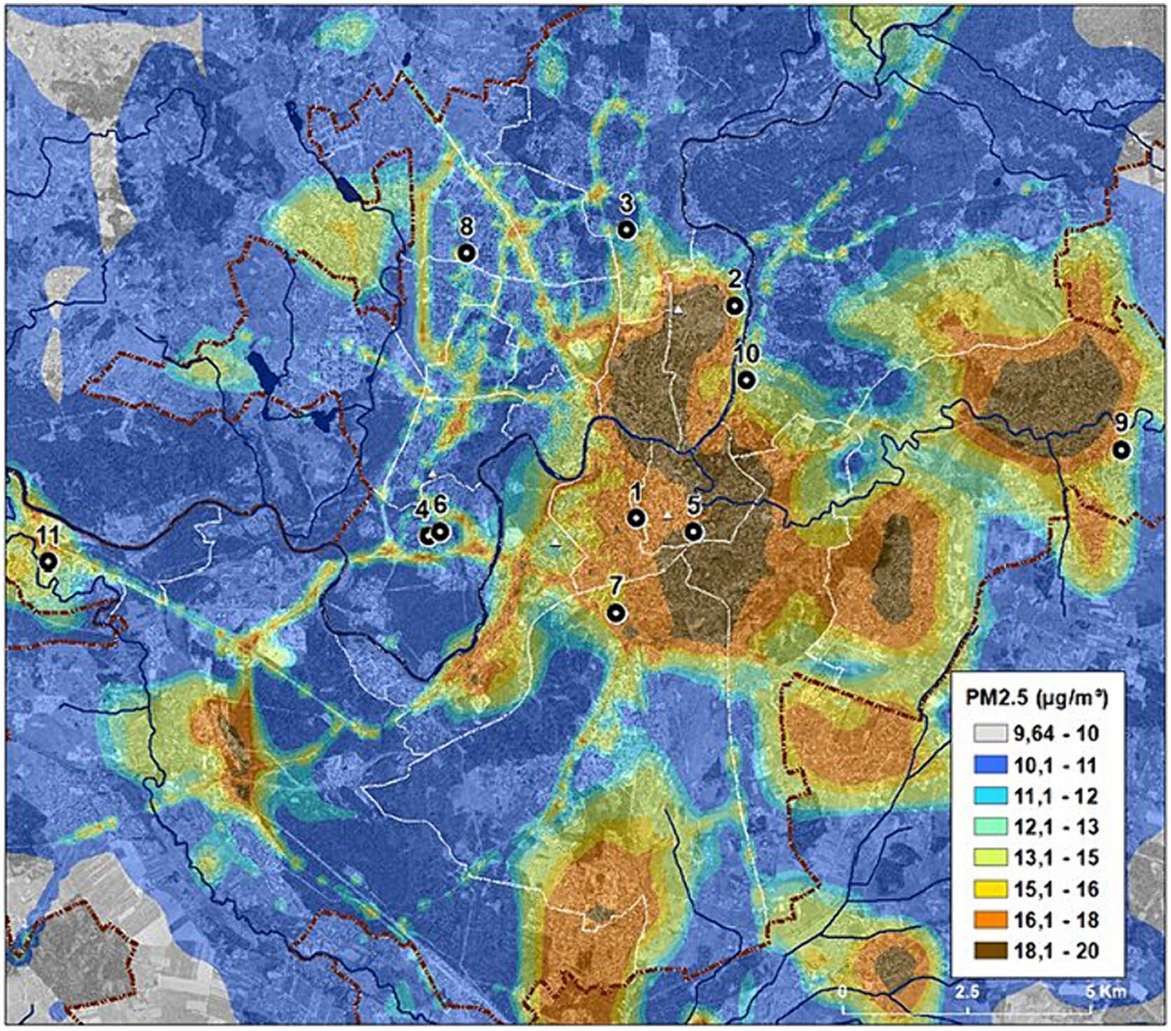
Location of the schools engaged in the research. The scheme of average annual ambient PM_2.5_ concentrations in Vilnius on 2018. Adapted from the public domain “Air Pollution Dispersion Maps” of the Environmental Protection Agency of Lithuania (19), published with permission, available at https://aaa.lrv.lt/lt/veiklos-sritys/oras/oro-uzterstumo-sklaidos-zemelapiai-duomenys-fonines-koncentracijos-paov-skaiciavimams/.

This study was carried out in Vilnius, Lithuania (54^o^41′17′′N, 25^o^15′8′′E). Primary school children (children in the age range of 6–11 years, grades 1–4) were enrolled. Invitations were sent to 107 Vilnius schools to participate in the study; 25 responded and agreed to participate. Every other school was randomly selected for inclusion in the study. One school did not have primary classes and was rejected. Finally, 11 schools were selected to participate in the study (Figure 1). A unique number was assigned for each school to protect the privacy of study participants. Schools numbered 1, 5, 7, and 10 were located in the downtown area, schools numbered 2, 3, 4, 6, and 8 were located in the peripheral part of the city, and those numbered 9 and 11 were located in the suburbs.

### 2.2 Collecting dust samples in schools

Filtering aerosol samples for trace element analysis in classrooms is known to be difficult, because collecting a significant sample mass can be a lengthy process. Therefore, we decided to replace the aerosol samples with dust samples, which usually accumulated in places inaccessible during cleaning in the classrooms. The adequacy of these samples is fairly obvious and saves considerable time. In this case, we obtained concentrations of microelements averaged over time via dust accumulation. Dust sampling in classrooms was taken from sites where dust accumulated: the surfaces of high cupboards (where aerosol particles are deposited due to coagulation) and behind central radiator heaters where aerosol particles are deposited due to thermophoretic forces. These natural dust aggregates have a purely aerosol origin and were collected mainly against the corners of the rear walls of radiator heaters. Other sources of dust such as particles brought into the classroom from the street with shoes were not included. The dust was collected using a vacuum cleaner on an analytical filter FPP type (Petryanov’s filters) in plastic boxes with a volume of 60 mL while tightly filled with dust.

### 2.3 Measurements of heavy metal concentrations in samples

Dust samples were crushed in a porcelain mortar and dried at 40°C until there was a steady mass in the drying chamber. Approximately 3 grams (g) of the dried sample was mixed and homogenized with 1 g of wax. The samples were then pressed into steel rings with a pressure of 15 kilonewtons (kN) to produce the final pellets. This preparation concentrated sample components and introduced some degree of homogeneity for improved analytical accuracy and precision. Samples were analyzed using a SPECTRO XEPOS (XEPOS HE) simultaneous ED-XRF spectrometer from SPECTRO Analytical Instruments. This model was equipped with an air-cooled, 50-W end-window X-ray tube—a bright laboratory-quality source optimized for maximum energy generation. The measurement time of one sample was 600 s, and the accuracy of elemental composition was less than 10%. The limit of detection (LOD) of trace elemental composition of samples is presented in Table 1. To further heighten stability, the excitation chamber, including the X-ray tube, optics, and detector, was maintained under constant vacuum conditions even in periods between measurements. The concentrations of Pb, W, Sb, Sn, Zr, Rb, Cu, Ni, Mn, Cr, V, As, Ba, Br, and Zn were thus measured in dust samples.

**Table 1:** Limit of detection (LOD) of trace element concentrations.

Element	Pb	W	Sb	Sn	Zr	Rb	Cu	Ni	Mn	Cr	V	As	Ba	Br	Zn
LOD, ppm	0.2	0.6	0.4	0.3	0.2	0.07	0.5	1.0	0.2	0.2	0.3	0.1	2.0	0.06	0.2

### 2.4 Data on morbidity in schools under study

The annual incidence of doctor-diagnosed acute infections of the upper respiratory tract (J00–J06) among 6- to 11-year-old pupils in each school was calculated based on clinical records of health care providers collected by the National Institute of Hygiene: acute nasopharyngitis (common cold) (J00), acute sinusitis (J01), acute pharyngitis (J02), acute tonsilitis (J03), acute laryngitis and tracheitis (J04), acute obstructive laryngitis and epiglottitis (J05), and acute upper respiratory infections of multiple and unspecified sites (J06). According to national legislation, personal codes of children and codes of diagnoses based on the Australian Modification of the International Statistical Classification of Diseases and Related Health Problems (ICD-10-AM) were received by the National Institute of Hygiene. The incidence of acute upper respiratory infections (J00–J06) per school represents an annual number of doctor-diagnosed cases among school children of 6–11 years of age divided by the total number of children of this age group in the school and multiplied by 100.

### 2.5 Statistical analysis

This study employed the linear regression model to determine the dependence of respiratory diseases on air pollution. The dependence of the annual incidence of respiratory infections on the microelemental concentration of dust samples can be expressed via a linear function where the dependent value is incidence (%), and the independent value is microelemental concentration (ppm).

We used Pearson’s correlation to evaluate the correlation between the elemental composition of dust samples and the incidence of acute upper respiratory infections in children. A *p*-value of < 0.05 was considered significant. IBM SPSS Statistics 23 was used for statistical analysis.

## 3 Results

### 3.1 The incidence of acute respiratory infections in children and elemental composition of dust samples

The incidence of upper respiratory infections per school ranged from 17.0% to 75.7%. The highest incidence was observed in Schools 9, 11, 6, and 10: 75.1%, 74.2%, 57.4%, and 52.2%, respectively. If we consider the incidence for the entire study period of 2016–2020, then the highest incidence rate was observed in 2019. This may be due to cross-diagnosis between influenza and upper respiratory tract infections (influenza cases were the highest in 2019) (20). Data on the elemental composition (Pb, W, Sb, Sn, Zr, Rb, Cu, Ni, Mn, Cr, V, As, Ba, Br, Zn) of dust samples in 11 schools in Vilnius are presented in Table 2.

**Table 2:** Elemental composition of dust samples, ppm.

School no.	Pb	W	Sb	Sn	Zr	Rb	Cu	Ni	Mn	Cr	V	As	Ba	Br	Zn
1	34	7.91	4.83	5.71	31.8	7.52	42.63	5.74	87	127.63	29.53	7.94	654	9.64	384
2	132	19.48	9.48	6.24	104	20.64	436.00	14.11	152	64	19.07	28.17	3,301	20.14	17,865
3	24	11.58	11.48	7.45	32	11.69	108.63	11.47	56	89	12.69	6.23	890	17.36	510
4	185	34.61	10.74	9.42	120	23.53	134.60	26.88	240	168	15.49	4.33	272	49.04	1887
5	504	14.69	13.00	9.42	93.55	18.52	77.83	20.46	159	180	17.18	20.93	1,653	19.07	1755
6	22	10.18	4.96	4.36	29.47	13.52	86.32	6.52	68	56.24	32.54	4.91	1,158	13.85	553
7	19	8.56	5.63	4.84	30.42	12.41	69.12	6.41	91	97.1	38.42	5.29	740	10.15	424
8	114	21.39	10.07	6.17	122	28.94	138.79	61.06	319	147	20.06	5.79	480	51.55	1,251
9	46	17.43	8.27	5.66	59	14.51	61.30	12.84	93	13.57	52.09	6.74	1923	16.92	247
10	17	11.85	7.52	6.11	27.42	11.81	134.12	8.4	72	96.21	41.63	6.29	1,532	14.93	319
11	14	9.85	4.56	5.09	31.25	7.42	91.59	5.78	86	108.52	41.09	6.32	984	11.39	248

**Figure 2:**
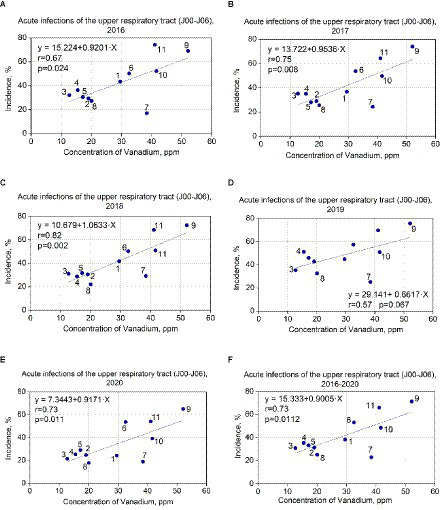
**(A–F)** Correlation between vanadium concentrations in dust samples and incidence of acute upper respiratory infections among pupils in studied schools.

Regression analysis was performed for all elements in Table 2. It turned out that a significant correlation was found only between vanadium concentrations and acute upper respiratory infections (J00–J06) in 2016, 2017, 2018, and 2020 years: r = 0.67, *p* = 0.024; r = 0.75, and *p* = 0.008; r = 0.82. *p* = 0.002; r = 0.73, and *p* = 0.01, respectively. In 2019 year, the correlation was not significant: r = 0.57, *p* = 0.067. The correlation between the concentration of vanadium and the average value of acute upper respiratory infections for the 2016–2020 years was also evaluated (r = 0.73, *p* = 0.01) because dust accumulation is an integral value for a longer time. The results are summarized in Figure 2. Linear regression equations were obtained and indicated reliable results. The linear regression data for the concentrations of vanadium (ppm) in dust samples and the incidence of acute upper respiratory tract infections in 11 schools (annual data) are presented in Table 3.

**Table 3:** Results of linear regression data for vanadium concentrations in dust samples and incidence of acute upper respiratory infections.

Model	Regression coefficient	Student’s *t*-test	*p*-value
Constant	15.224	1.421	0.189
Vanadium, ppm (Incidence for 2016)	0.920	2.717	0.024
Constant	13.722	1.529	0.161
Vanadium, ppm (Incidence for 2017)	0.954	3.361	0.008
Constant	10.679	1.371	0.204
Vanadium, ppm (Incidence for 2018)	1.063	4.319	0.002
Constant	29.139	2.898	0.018
Vanadium, ppm (Incidence for 2019)	0.662	2.082	0.067
Constant	7.344	0.805	0.442
Vanadium, ppm (Incidence for 2020)	0.917	3.177	0.011
Constant	15.333	1.712	0.121
Vanadium, ppm (Incidence for 2016–2020)	0.900	3.180	0.011

For vanadium data in 2016 (in case of incidence of acute upper respiratory infections), the F-statistic is 7.381, the *p*-value is 0.024, and the coefficient of determination (R^2^) is 0.45 (for the 2016 year). The F-statistic is 11.293, the p-value is 0.008, and R^2^ is 0.56 for 2017. The F-statistic is 18.661, the p-value is 0.002, and R^2^ is 0.68 for 2018. The F statistic is 4.332, the p-value is 0.067, and R^2^ is 0.32 for 2019. The F-statistic is 10.096, the *p*-value is 0.011, and R^2^ is 0.53 for 2020. The F-statistic is 10.114, the p-value is 0.011, and R^2^ is 0.52 for the average of 2016–2020.

Thus, *p*-values are less than 0.05, which indicates coefficient reliability. The coefficients of determination (R^2^ = 0.45, R^2^ = 0.56, R^2^ = 0.68, R^2^ = 0.53, R^2^ = 0.52) demonstrate that the vanadium data (in the case of incidence of acute upper respiratory infections) correspond to the linear regression. We checked that our linear regression assumptions concerning the residual are satisfied. The residuals are normal (Kolmogorov–Smirnov test), and their mean does not differ from 0. The summary of the results is presented in Figures 2A–F.

According to the linear regression equation, an increase in the concentration of vanadium by 1 ppm leads to an increase in the incidence of acute upper respiratory infections by 0.92–1.06% in 2016, 2017, 2018, and 2020 (Figures 2A–C,E). The exception is 2019 (0.66%; Figure 2D) possibly due to the highest incidence of influenza in that year. It can be caused by cross-diagnosis between influenza and upper respiratory tract infections—especially in cases where a viral search was not conducted.

## 4 Discussion

This is the first study devoted to the relationship between the chemical composition of natural dust aggregates and respiratory morbidity in children. Trace element analysis can indicate the time-dependent pollution history of public buildings or residential areas. It allows us to evaluate the concentration of heavy metals in pre-aggregate aerosol forms of pollutants. A significant correlation was found between the concentration of vanadium in dust samples and physician-diagnosed upper respiratory infections in younger school-age children. The concentrations of vanadium in Vilnius schools were 12.69–52.09 ppm.

Vanadium is important for normal cell function and development (21, 22). It exists in all tissues involved in glucose homeostasis, lipid metabolism, and antioxidant functions. For the general population, food is the major source of vanadium exposure (23–25). Despite the positive effect of vanadium on various processes in humans, a safe dose is still unclear. High concentrations can lead to various pathological alterations including modified intracellular enzyme systems, which have an impact on digestion, respiration, etc. (26–29).

Two-thirds of vanadium enters in aerosol form due to anthropogenic sources, such as the burning of fossil fuel, vehicle emissions and leaks when used it in industrial production (30). Therefore, it is not surprising that the maximum concentrations of vanadium in dust samples are seen in Schools No. 9, 10, and 11. School No. 9 is located in a suburb of Vilnius where there are 20 industrial enterprises and many houses use stove heating. School No. 10 is located next to a very busy transport artery. School No. 11 is located near a big paper factory. The children in School No. 6 are in the zone of influence of the thermal power plant when the wind is from the South.

Vanadium is especially dangerous in the form of aerosols. Only <1–2% of vanadium is absorbed by the body during oral ingestion (23), but up to 90% of vanadium is absorbed when inhaled (31, 32). At the same time, the greatest harm comes from aerosols carrying vanadium with sizes below 2.5 microns, because these particles can reach the alveolar sacs. Prolonged inhalation of vanadium aerosols can cause shortness of breath, wheezing, cough, epistaxis, headache, dizziness, fatigue, as well as cancer (29, 33). One hour per day exposure of rabbits to vanadium of 20–40 mg/m^3^ during several months caused chronic rhinitis, tracheitis, emphysema, atelectasis, and bronchopneumonia (34). Continuous exposure to a concentration of 10–30 mg/m^3^ was distinctly toxic to rabbits, causing bronchitis and pneumonia, loss of weight, and bloody diarrhea. With rats, 10 mg/m^3^ was toxic, and a small exposure of 3–5 mg/m^3^ caused the same symptoms with 2 months delay. A lethal exposure was considered to be 70 mg/m^3^ if prolonged for more than 20 h. In another study, groups of 50 male and 50 female F344 rats were exposed to 0, 0.5, 1, or 2 mg vanadium pentoxide/m3 (0, 0.28, 0.56, and 1.1 mg vanadium/m^3^) 6 h/day, 5 days/week for 104 weeks. Alveolar histiocytic infiltrates were observed in males and females rats exposed to ≥0.28 mg vanadium/m^3^ (35). An inhalation model demonstrated that vanadium generates histological and physiological changes in different cells and organs including the lung, lymphoid organs, and the immune system. Oxidative and nitrosative stress play a relevant role in the vanadium toxic mechanisms (30, 36).

The U.S. Environmental Protection Agency (EPA) published a review (37) that summarized 67 human studies devoted to the health effects of vanadium on infants, children, pregnant women, the general population (adults) and occupational workers. The predominant health outcomes investigated included respiratory, cardiovascular, nervous system and immune system effects. Twenty studies examined the effects of vanadium on the respiratory system; however, only two of these (38, 39) were devoted to children’s respiratory health. Patel et al. reported that increases in ambient vanadium concentrations (0.0033 mg/m^3^) were associated significantly (*p* = 0.0003) with an increased probability (31%) of wheezing in children through age two (38). Gehring et al. did not find an association between vanadium in PM_2.5_ or PM_10_ and children’s respiratory health (39). However, PM constituents, in particular iron, copper and zinc, may increase the risk of asthma and allergies in schoolchildren. Five studies (40–44) were devoted to general population-adult respiratory health and 13 studies were on occupational exposure and respiratory health. An association has been reported between hospitalization for respiratory illness and observed vanadium concentrations in PM_2.5_ outdoors (41). Urinary vanadium, nickel, and antimony have been reported to be associated with increased exhaled nitric oxide (42). Zenz and Berg studied responses in nine human volunteers exposed to vanadium pentoxide from 1 to 0.2 mg/m^3^ (particle size, 98% < 5 μm) for 8 h in a controlled environmental chamber (40). Coughing began and then remained in all subjects for 7 to 10 days. However, Lagorio et al. (43) and Wu et al. (44) found no adverse effects of vanadium on the adult respiratory system. There is even more limited and controversial data on the disease initiation and/or modification effects of vanadium in children (37, 45).

In our study, a significant and replicable correlation was found between the concentration of vanadium in the samples of natural dust aggregates and the incidence of doctor-diagnosed acute upper respiratory infections in younger school-age children in the years 2016–2020. The exception was 2019, when a moderate, but non-significant correlation was found. It can be caused by a very high incidence of influenza cases and misdiagnosis of other than influenza respiratory infections especially when the diagnosis was done without virus verification. The highest incidence of influenza among children in the period of 2016–2020 was observed in 2019.

There are a limited number of studies on the microelemental composition of aerosol particles in residential premises (46–49) and schools (50–52). In these cases (46, 48, 49), indoor dust does not characterize the composition of the aerosol particles because the dust can be of various origins. As a rule, the dust was collected by a vacuum cleaner from the floor surfaces. One study was conducted in kindergartens (53), but it did not target the composition of the aerosols, and dust was collected from floors and window sills.

Studies of the microelemental composition of aerosol particles is a very laborious process. This also applies to the time of sampling when using special filters, i.e., from 8 h to several days or even months. To date, this method has been used to determine the composition of aerosols (47, 50–52). This challenging analysis likely explains the paucity of data on the elemental composition of aerosol pollution. Indeed, data about the microelemental composition of aerosol particles and their relation to diseases in children are rare and insufficient (54–57).

High PM_2.5_ values in residential areas have been associated with lower lung function among urban children. An association was found between short-term vanadium exposure and DNA methylation in the asthma gene loci (58). A study of children (10–12 years of age) living in the vicinity of a facility involved in hydrometallurgical processing of vanadium-rich slag found significant decreases in lymphocyte stimulation with phytohemagglutinin and an increase in the incidence of viral and bacterial respiratory infections (59).

Data from occupational studies suggest that the lowest-observed-adverse-effect level of vanadium is assumed to be 20 μg/m^3^, based on chronic upper respiratory tract symptoms (60). Particularly high PMC values (up to 230 μg/m^3^) were earlier reported in the air of classrooms School No. 9 (61), while vanadium concentration in dust samples from the same school was as high as 52 ppm. This allows us to calculate the hypothetic amount of inhaled vanadium before it turns to dust aggregates. Thus, in the case of inhalation of 1 m^3^ of air, 230 μg of particulate matter including 0.012 μg of vanadium can enter the respiratory tract of a child. Taking into account one school year (39 weeks, 5 days per week, and 6 h per day) and an inhalation rate of 0.9 m^3^/h (62), that is 1,053 m^3^/year, and we can estimate that the annual inhaled amount of vanadium can be 12.64 μg. However, we do not know the proportion of inhaled vanadium that has settled in the lungs.

Studies of the effects of heavy metals on the development of respiratory morbidity in children (with different levels of microelemental concentrations in polluted air) are rarer and more controversial (37, 45, 59). The EPA emphasizes the need for more in-depth research on vanadium to identify the mechanism of this microelement’s effects on human health and the possibility of determining safe concentrations. It seeks to analyze the impact of short-term and long-term cumulative exposures (37).

Nevertheless, we hypothesize that vanadium-induced damage to the respiratory system may be complex. These may include the destruction of the barrier function of the bronchial wall due to chronic inflammation, reduced clearance of bacteria in the lungs, and reduced capacity of alveolar macrophages. It has been earlier reported, that exposure of human fibroblasts to vanadate effectively causes DNA strand breaks, and co-exposure of cells to other genotoxic agents may result in persistent DNA damage (24). We can also extrapolate our suggestions from brake abrasion dust (BAD) research which shows the dose-dependent relationship between BAD and the impaired ability of immune cells to ingest respiratory pathogens and enhanced inflammatory signaling in a transient but metal-dependent manner (63). Responses to particles were characterized by decreased mitochondrial depolarization, increased secretion of IL-8, IL-10 and TNF-α as well as decreased phagocytosis of *S. aureus*. Mitochondrial dysfunction leading to early airway remodeling was recently identified in preschool children with infection-induced recurrent wheezing syndrome (64).

The World Health Organization (WHO) has no data on the annual exposure level of children and adults to vanadium (60). However, WHO considered annual average values for inhalation of vanadium for urban areas to be in the range of 0.05–0.18 μg/m^3^. In the most densely populated areas, maximum vanadium concentrations reach up to 2 μg/m^3^. In Lithuania, the maximum permissible indoor concentration of vanadium for both adults and children is 1 μg/m^3^ (27). In the present study, we found a linear relationship between respiratory morbidity in children and the concentration of vanadium in dust aggregates. This suggests that any concentration of vanadium inhaled by children can increase respiratory morbidity caused by viruses and bacteria. It supposes the necessity to re-evaluate the current understanding of the safe limits of inhaled vanadium in children.

There are some limitations of our study. Eleven schools participating in our survey might be insufficient to analyze all health-damage effects of pollutants. In contrast to vanadium, the concentrations of other heavy metals in dust samples did not reach the threshold values, and a correlation with the incidence of upper respiratory infections was not found. Thus, the disease initiation and/or modification effects of these heavy metals need a larger research sample. Another limitation is related to an unknown period of dust accumulation in one school or another. The incidence of respiratory infections was calculated based on medical records only and some home-cared mild respiratory infections can be missed or improperly recorded.

Our research is the first attempt to evaluate the impact of the chemical composition of indoor air pollutants on the respiratory morbidity of younger school-age children using samples of natural dust aggregates taken from primary schools. It opens the possibility for further prospective studies with the evaluation of known periods of dust accumulation and dose–response effects of different pollutants.

## 5 Conclusion

The incidence of acute upper respiratory infections among 6- to 11-year-old children is related to the concentration of vanadium in the natural dust aggregates collected from their primary schools. Unlike the difficulties of aerosol sampling using filters, samples of natural dust aggregates collected in classrooms are very convenient and reliable materials for studying the time-dependent trace element composition of indoor pollutants. Regular assessment of indoor air quality including the post-aerosol dust aggregates can be an important tool for the prevention and control of respiratory morbidity in children.

## Data availability statement

The original contributions presented in the study are included in the article/supplementary material, further inquiries can be directed to the corresponding author.

## Author contributions

NP: Writing – review & editing, Writing – original draft, Visualization, Methodology, Formal analysis, Conceptualization. VT: Software, Writing – review & editing. LaV: Formal analysis, Writing – review & editing. IJ: Investigation, Writing – review & editing. VS: Investigation, Writing – review & editing. IV: Formal analysis, Writing – review & editing. VV: Validation, Writing – review & editing. RV: Investigation, Writing – review & editing. AlV: Investigation, Writing – review & editing. TA: Formal analysis, Writing – review & editing. LuV: Writing – review & editing. MB: Formal analysis, Writing – review & editing. JN: Software, Writing – review & editing. NT: Writing – review & editing, Writing – original draft, Methodology, Conceptualization. ArV: Supervision, Writing – review & editing, Writing – original draft, Data curation, Conceptualization.
